# Minutes of the Annual Meeting of the Ohio Dental College Association

**Published:** 1854-04

**Authors:** 


### *Minutes of the Annual Meeting of the Ohio Dental College Association.*

The Society came to order at 2 o'clock P. M., of Monday, February 20th, 1854, at the College, with the President in the chair.

On calling the roll, the following members were found to be present, viz.: Drs. James Taylor, Bonsall, J. B. Smith, H. R. Smith, Taft, Allen, Duncan, and Berry.

The minutes of last meeting being first read, the President informed the Society that the recommendations made to the Board of Trustees, last year, in relation to the Professor's chairs, had been carried out by them, and that the Faculty now consisted of Drs. James Taylor, Thos. Wood, J. B. Smith, H. R. Smith, and J. Allen.

Also reported that the committee on improvements had not expended any money within the last year, only having had such improvements made as the Faculty were willing to pay for.

Also, that an insurance had been effected on the building, and the taxes paid, and that there was now due to the Association from the Faculty a balance of about $368, on account of rent of the building for the last two years.

He also reported that all the stock which remained untaken last year, had been subscribed for.

A letter was received from Dr. Peebles, Lexington, Mo., informing of his inability owing to sickness, to attend the meeting this year, which was cause of general regret.

Dr. Taylor handed in some proposals for building a new college edifice, and suggestions connected therewith, which, on motion of Dr. Taft, were laid over till 9 A. M., to-morrow, to which time the Association adjourned.

*Second Day—Morning Session.*—Met at 9 A M., pursuant to adjournment.

Present—Drs. Taylor, Taft, Bonsall, H. R. Smith, Allen, A. S. Tulbert, Berry, and J. B. Smith.

The minutes of yesterday were read and adopted.

Drs. Sam. Wardle, J. M. Brown and Ebenezer Bray, having each subscribed for one share of stock, were balloted for, and each elected a member of this Association,

The matter of rebuilding the College now coming up, and considerable discussion having been had and information elicited as to the present pecuniary condition of the College, and its future prosperity, it was, on motion of Dr. Berry—

*Resolved*, That the Board of Trustees be, and they are hereby authorized to increase the number of shares of stock from thirty-eight, which it is at present, to fifty.

Also, on motion of Dr. Bonsall, it was—

*Resolved*, That the Trustees be, and they are hereby authorized to execute, if necessary, a mortgage on the new college building for the purpose of raising funds sufficient to liquidate any debt incurred in the erection of the same.

And, on motion of Dr. Berry, the Association went into the appointment of a building committee, as follows, Drs. James Taylor, Bonsall, and Berry, who were authorized to draw on the treasurer for such moneys as they may require.

Drs. Berry and Bonsall were now appointed to fill the vacancies in the examining committee, occasioned by the absence of Drs. Peebles and Goddard.

Dr. Jno. Allen having tendered his resignation as Professor of Operative Dentistry as follows:

" *To the Trustees of the Ohio College of Dental Surgery.*

" Gentlemen—As it becomes necessary, from a change in my business operations, to be absent from this city during the annual session of the Institution; and being also desirous of promoting the best interests of the school, I most respectfully tender you my resignation as a member of your Faculty, in order that the chair which I have had the honor to fill for the last three years, may now be filled by another; and I would here take occasion to acknowledge my grateful feelings for the marked respect, attention, and forbearance, which have ever been shown me during my connection with the Institution.

" Yours respectfully,

" *Cincinnati, Feb.* 21, 1854. Jno. Allen."

It was, on motion of Dr. Berry—

*Resolved*, That the resignation of Dr. Allen, as a Professor in the Ohio College of Dental Surgeons, having been tendered to the Board of Trustees, the stockholders hereby present him their heartfelt thanks for the efficient and faithful manner in which he has discharged the duties devolving on the chair which he occupied.

On motion of Dr. H. R. Smith, the nomination of a suitable person to fill the vacancy was postponed till 4 P. M., this day, when it shall be the order of business.

Dr. Taylor reported the class of the present winter to consist of twenty-three.

Adjourned till 4 P. M.

*Afternoon Session.*—Met at 4 P. M.

The minutes of the morning session were read and adopted.

On motion of Dr. Talbert, went into an election, and it appeared that Dr. Taft, of Xenia, Ohio, was nominated to the Board of Trustees to fill the chair vacated by Dr. Allen.

On motion, went into an election for officers of the Association for the ensuing year, when the following gentlemen were found to be elected, viz.

Dr. James Taylor, *President.*
" H. R. Smith, *First Vice-President.*
" A. Berry, *Second* " "
" Chas. Bonsall, *Secretary.*
" J. B. Smith, *Treasurer.*

Drs. Ullery, Hamill, and Quinland, Dental Examiners; and Drs. L. M. Lawson and John Davis, Medical Examiners.

Adjourned till 9 A. M., to-morrow.

*Third Day—Morning Session.*—Met at 9 A. M.

Present—Drs. James Taylor, Berry, H. R. Smith, Talbert, Van Emon, Taft, Allen, Goddard, Bonsall, and J. B. Smith.

Yesterday afternoon's minutes were read and adopted.

Drs. M. De Camp, of Mansfield, Ohio, J. W. Baxter, of Warsaw, Ky., A. S. Dwyer, of Louisville, Ky., and E. A. Herman, of Nashville, Tenn., being now proposed as members, were each balloted for, and duly elected as members of this Association, and signed the constitution.

A plan for the new college building being now presented by the building committle, it was, on motion of Dr. H. R. Smith—

*Resolved*, That the building committee be authorized to erect, on the site of the present building, a suitable edifice, with a stone front (if possible,) not to exceed in cost five thousand dollars.

On motion of Dr. Taft, the building commitee were authorized to proceed immediately to the erection of said building, and all arrangements were left discretionary with them.

Adjourned till 9 o'clock A. M. to-morrow.

*Fourth Day—Morning Session.*—Met pursuant to adjournment—when the minutes of the previous meetings were read, and adopted as a whole, and the Secretary requested to furnish a copy for publication in *The Dental Register of the West.*

On motion, adjourned to meet in the new college edifice on Monday, February 21st, 1855, at 2 o'clock, P. M.

CHARLES BONSALL,
*Secretary.*

NOTE.—It may perhaps be proper to mention, in reference to the action of the Society in relation to building, that of those stockholders who were not present, a large majority were represented by proxy.—ED.

